# Outbreak of Multi-Drug Resistant *Pseudomonas aeruginosa* Bloodstream Infection in the Haematology Unit of a South African Academic Hospital

**DOI:** 10.1371/journal.pone.0055985

**Published:** 2013-03-14

**Authors:** Maanda Mudau, Rachael Jacobson, Nadia Minenza, Lazarus Kuonza, Vida Morris, Heather Engelbrecht, Mark P. Nicol, Colleen Bamford

**Affiliations:** 1 Centre for Tropical, Opportunistic and Hospital Infections, National Institute for Communicable Diseases, Johannesburg, South Africa; 2 Division of Medical Microbiology, University of Cape Town, Cape Town, South Africa; 3 South African Field Epidemiology & Laboratory Training Programme, Johannesburg, South Africa; 4 School of Health Systems & Public Health, Faculty of Health Sciences, University of Pretoria, Pretoria, South Africa; 5 Provincial Government of Western Cape Department of Health, Cape Town, South Africa; Vrije Universiteit Brussel, Belgium

## Abstract

**Objective:**

To describe an outbreak of multi-resistant *Pseudomonas aeruginosa* bloodstream infections (MRPA-BSI) that occurred in the haematology ward of a tertiary academic hospital in Cape Town, South Africa, and determine risk factors for acquisition of MRPA-BSI.

**Methods:**

The outbreak investigation included a search for additional cases, review of patient records, environmental and staff screening, molecular typing using pulsed-field gel electrophoresis (PFGE) and Multi-locus sequencing (MLST) and a retrospective case-control study.

**Results:**

Ten MRPA-BSI cases occurred in the haematology ward between January 2010 and January 2011. The case fatality rate was 80%. Staff screening specimens were negative for MRPA and an environmental source was not identified. PFGE showed that 9/10 isolates were related. MLST showed that 3 of these 9 isolates belonged to Sequence type (ST) 233 while the unrelated isolate belonged to ST260.

**Conclusion:**

We have described an outbreak of MRPA-BSI occurring over an extended period of time among neutropenic haematology patients. Molecular typing confirms that the outbreak was predominantly due to a single strain. The source of the outbreak was not identified, but the outbreak appears to have been controlled following intensive infection control measures.

## Introduction

The burden of nosocomial infection in low and middle income countries has been under-appreciated in the past [1], with the extent and impact of infections due to multi-resistant organisms such as *Pseudomonas aeruginosa*, in particular being under-estimated [2]. In the absence of well-resourced surveillance systems, outbreak reports can serve to highlight serious pathogens. While such outbreaks are frequently reported in the published literature, relatively few of these reports originate from low income countries.

*P. aeruginosa* is a common cause of infection among hospitalized patients. It is inherently resistant to certain antibiotics due to a variety of resistance mechanisms. Treatment is further limited by the ability of the organism to rapidly develop additional resistance during treatment [3], [4]. Risk factors for *P. aeruginosa* infection include presence of indwelling devices, admission to an intensive care unit, prior antibiotic use, length of hospitalization, severe underlying disease and impaired immunity [5], [6].

We investigated an outbreak of *P. aeruginosa* bloodstream infection that occurred in the clinical haematology intensive care unit of a tertiary academic hospital in Cape Town, South Africa. Our report highlights the infection control, and general public health challenges, posed by antimicrobial-resistant organisms in resource-limited settings.

## Methods

### Setting

The haematology ward of the tertiary academic hospital is a shared intensive care unit (ICU) with 12 isolation (private) rooms, of which 6 are used by a public sector hospital and 6 by a private hospital. Each room has an attached bathroom, as well as a nursing anteroom. All have High-Efficiency Particulate Air (HEPA) filters and 5 have a laminar flow system installed to provide protection against airborne pathogens. Access to the ward is controlled and strict infection control procedures are followed.

Each hospital has its own nursing staff. However, various facilities, including the nurses' station and sluice room, as well as staff facilities, such as tea room and change rooms, are shared by the two hospitals. Food is provided to all patients from the kitchen of the private hospital. Medical staff and cleaning staff attached to the public hospital provide services to both groups of patients. In addition to the regular ward staff, temporary nursing staff from nursing agencies is sometimes employed. The ward admits patients with malignant and non-malignant haematological conditions, most of whom have undergone haemopoetic stem cell transplant (HSCT) or are undergoing intensive chemotherapy. These patients are usually highly immuno-compromised and are extremely susceptible to infections. Patients are confined to individual rooms, but do occasionally leave the ward, e.g. to visit the radiology department for investigations.

Differences between the private and public sections of the unit that might impact on the risk of infection include the better nurse: patient ratio in the private sector, the more liberal patient selection criteria in the private sector permitting the treatment of older patients and the use of filgrastim, a granulocyte colony-stimulating factor analogue, to shorten the period of neutropenia post- stem cell transplant in selected high-risk patients in the private sector.

### Data Collection

The laboratory database was searched to identify all *P. aeruginosa* isolates from blood culture specimens from 1 January 2009 until 31 January 2011. Patients with *P. aeruginosa* isolates found in blood specimens collected during their admission in the haematology ward were identified. The hospital information system and patient folders were reviewed in order to determine the hospitalization history of the cases, demographic characteristics and clinical history. Since the study was conducted as an outbreak investigation, informed consent of patients was not sought on the grounds that a) clinical information was collected solely by retrospective record review b) no additional specimens were collected from patients for study purposes c) all patient identifying data was removed prior to dissemination of results. The study was approved by the Human Research Ethics Committee of the University of Cape Town (Ref: 393/2011) and conducted according to the ethical guidelines and principles of the International Declaration of Helsinki and the Medical Research Council (MRC) guidelines for Good Clinical Practice (GCP).

### Staff and Environmental Screening

In November 2010 after a series of meetings and consultations staff members were requested to submit stool specimens to investigate gut colonization with *P. aeruginosa* and to report any other possible personal sources of MRPA colonization such as chronic otitis externa or any infection around finger nails. Additional samples, such as contact plates from hands that might detect more transient carriage, were not sought from staff because of the delay in detection of the outbreak. Informed consent from staff was not sought as screening of staff for possible carriage of the organism of interest is good clinical practice and an essential part of a public health intervention in this type of outbreak investigation. No specific therapy would have been indicated for staff found to be carrying resistant *P. aeruginosa*. However, they would have been redeployed to other areas of the hospital temporarily. The employers undertook that no staff member would be financially or otherwise disadvantaged as a result of testing. No penalties were applied to staff who did not submit specimens.

Because of concerns around the safety of cleaning procedures and because of *P. aeruginosa's* propensity to survive in moist environments, initial sampling in October 2010 focused on cleaning equipment, water samples and swabs from taps in the patient isolation rooms and from water used in respiratory therapy equipment. Environmental surfaces were swabbed with sterile swabs, pre-moistened in sterile water. Swabs were placed in commercial transport medium and transported rapidly to an on-site laboratory. For collection of water samples, 100 ml of water was collected directly into a commercially-available sterile bottle containing sodium thiosulphate powder for immediate neutralization of residual chlorine.

### Microbiological Testing

Microbiology testing was carried out at an on-site accredited microbiology laboratory. The laboratory uses the Bactec 9000 blood culture system (Becton Dickinson, New Jersey), while other specimens are inoculated onto a selection of appropriate agar media. Screening specimens were plated onto McConkey agar supplemented with 4 µg/ml of gentamicin. *P. aeruginosa* was identified using Vitek 2 (BioMerieux, North Carolina) Gram negative card, supplemented as necessary by phenotypic tests such as oxidase positivity and production of green pigment. Antimicrobial susceptibilities were determined by the Vitek 2 (BioMerieux, North Carolina), using the AST-N133 card and interpreted according to Clinical Laboratory Standards Institute (CLSI) criteria [7].The following antibiotics were tested: piperacillin-tazobactam, ceftazidime, cefepime, meropenem, imipenem, ciprofloxacin, gentamicin, amikacin and colistin. Water samples were processed at the local accredited public health laboratory, according to a standard protocol for detection of *P. aeruginosa* based on membrane filtration, incubation of the membrane filter in thioglycolate broth for 24 h, and subsequent subculture and identification [8].

Pulsed-field gel electrophoresis was performed and restriction endonuclease digestion of the intact genomic DNA of all isolates was performed *in situ* using SpeI [9]. Restriction fragments of DNA were separated on a 1% wt/vol agarose gel using a CHEF-DRII GeneNavigator apparatus (Amersham Biosciences, Fairfield, CT, USA) at a pulse time of 5 to 30 s for 20 h at 200 V in 0.5% vol/vol TBE buffer maintained at 14°C. Following staining with ethidium bromide (0.5 µg/ml), the DNA fingerprints were visualized and photographed under UV light. DNA fragment analysis was performed using GelCompar II version 5.1 (Applied maths, St-Martens-Latem, Belgium) and clusters were defined using the Dice coefficient of similarity. Dendrograms were drawn with a position tolerance of 1% and optimization of 1%. A cluster of isolates was defined to include all isolates at greater than 80% similarity in their DNA profiles according to the criteria of Tenover [10].

Multi-locus sequencing (MLST) of seven housekeeping genes (acetyl coenzyme A synthetase *acs*A, shikimate dehydrogenase *aro*E , GMP synthase *gua*A , DNA mismatch repair protein *mut*L, NADH dehydrogenase I chain C, D *nuo*D, phosphoenolpyruvate synthase *pps*A and anthranilate synthetase component I *trp*E) was performed on selected isolates spanning the outbreak period according to a previously described method for *P. aeruginosa* [11]. The MLST database was used to determine allelic profiles for each gene, and the subsequent sequence type, for each of the strains (http://pubmlst.org/paeruginosa/).

### Case-Control Study

A retrospective case-control study was conducted to identify potential risk factors for *P. aeruginosa* bloodstream infection among patients admitted to the unit. Cases identified between 1 December 2009 and 31 January 2011 were compared to patients admitted to the unit over the same period of time who had bloodstream infections due to Gram-negative bacteria other than *P. aeruginosa* (GN Controls) and to patients who never developed bloodstream infection due to Gram-negative bacteria (Non-GN Controls).

Specific variables that were studied included age, sex, hospital (public or private), length of hospitalization, primary diagnosis at admission, antibiotic use, haemopoetic stem cell transplant (HSCT) and chemotherapy. Antibiotic use was defined as antibiotics used in the 30 days prior to isolation of *P. aeruginosa* for cases and during any episode of admission in the ward for controls. HSCT was defined as within one year prior to isolation of *P. aeruginosa* for cases and within one year prior to final discharge from the ward for controls. Chemotherapy was defined as any chemotherapy given during the index admission prior to isolation of *P. aeruginosa* for cases and chemotherapy during any episode of admission in the ward for controls.

### Statistical Analysis

Data management and statistical analysis was done using STATA Intercooled version 11 (StataCorp. 2009. *Stata Statistical Software: Release 11*. College Station, TX: StataCorp LP). Continuous and discrete variables were summarized by median and interquartile range and were compared between the cases and controls using the Mann-Whitney U test. Categorical variables were summarized by proportions (percentages) and Fischer's exact test was used to test for statistical significance. Odds ratios and their 95% confidence intervals were calculated. Fischer's exact test was used to test for statistical significance. Backward elimination stepwise logistic regression analysis (P-removal: 0.05) was used to identify independent risk factors for *P. aeruginosa* infection. Variables that were significantly associated with MRPA-BSI at p<0.25 significance level were considered for possible inclusion in the logistic regression model.

## Results

### Description of the outbreak

This outbreak of MRPA-BSI was detected in October 2010 after two patients who were admitted to the clinical haematology ICU died very rapidly of MRPA-BSI over one weekend. Both patients had been hospitalized for prolonged periods of time and were neutropenic. The initial response to the outbreak was a review of all practices in the ward, specifically maintenance of the environmental infection control system and adherence to basic infection control procedures. In November another three patients developed *P. aeruginosa* bloodstream infection, two of whom died. The ward was closed in the first week of December and thoroughly cleaned. After reopening one further patient developed MRPA-BSI and died in January 2011. Four additional cases of MRPA-BSI that had occurred prior to detection of the outbreak were identified through a retrospective search of the laboratory database. There were no reported cases of MRPA infection or colonisation in the unit prior to January 2010.

Eight of the cases were female (80%). All the cases were neutropenic at the time of collection of the first blood culture with MRPA. Seven (70%) of the cases had an admission diagnosis of Acute Myeloid Leukemia (AML), one had Acute Lymphoblastic Leukemia (ALL), one had Aplastic Anaemia and one with a B-cell Lymphoma. Eight of the patients died (CFR = 80%), all within 48 hours of development of MRPA-BSI and despite the initiation of colistin therapy in some patients (given usually at a dose of 3 million units 8 hourly) Most of the patients had prolonged periods of hospitalization (median = 61 days) prior to development of MRPA-BSI. Table 1 gives details of individual patients.Two patients had evidence of MRPA colonisation at other sites, one (case 9) on a skin swab taken from the site of insertion of a vascular catheter four days prior to development of BSI, and one (case 10) from a stool sample taken simultaneously with the detection of BSI. Although weekly stool samples were taken for surveillance purposes from all patients admitted to the haematology ICU, no other patients were colonised with MRPA during the period of the outbreak (December 2009 to January 2011).

**Table 1:** Characteristics of patients with *Pseudomonas aeruginosa* bloodstream infection in a haematology intensive care unit of a tertiary academic hospital in Cape Town, South Africa, January 2010–January 2011.

Patients	Hospital	Age	Sex	Diagnosis	Length of hospitalisation in unit (days)	Date of infection	Outcome
**Case 1**	public	46	M	AML	11	03/01/2010	Died
**Case 2**	private	16	M	Aplastic Anaemia	52	28/01/2010	Discharged
**Case 3**	public	28	F	B-cell Lymphoma	70	31/05/2010	Died
**Case 4**	private	58	F	AML	74	23/06/2010	Died
**Case 5**	private	20	F	ALL	105	06/10/2010	Died
**Case 6**	public	37	F	AML	19	08/10/2010	Died
**Case 7**	private	48	F	AML	29	05/11/2010	Died
**Case 8**	private	46	F	AML	49	21/11/2010	Died
**Case 9**	public	60	F	AML	126	23/11/2010	Discharged
**Case 10**	public	20	F	AML	125	13/01/2011	Died

AML Acute Myeloid Leukemia ALL Acute Lymphoblastic Leukemia.

One hundred and eighteen patients were admitted to the haematology unit between 1 December 2009 and 31 January 2011 generating a total of 4399 patient-days. Of these patients 26 developed BSIs with Gram-negative bacilli other than *P. aeruginosa*.

### Microbiology Results

In 9 cases the initial *P. aeruginosa* isolate was resistant to ampicillin, cefuroxime, ceftriaxone, cefepime, ceftazidime, co-amoxiclav, piperacillin-tazobactam, ertapenem, meropenem, imipenem gentamicin, amikacin, cotrimoxazole, ciprofloxacin and tigecycline. Two of these 9 isolates had reduced susceptibility to colistin, with MICs of 4 and 8 µg/ml, respectively. The remaining patient (case 2) had multiple isolates over time, with the initial 3 isolates being susceptible only to amikacin, ceftazidime and colistin. Two subsequent isolates from this patient demonstrated a progressive increase in resistance, with the first being susceptible only to ceftazidime and colistin, and the final isolate being susceptible to colistin only.

**Figure 1:**
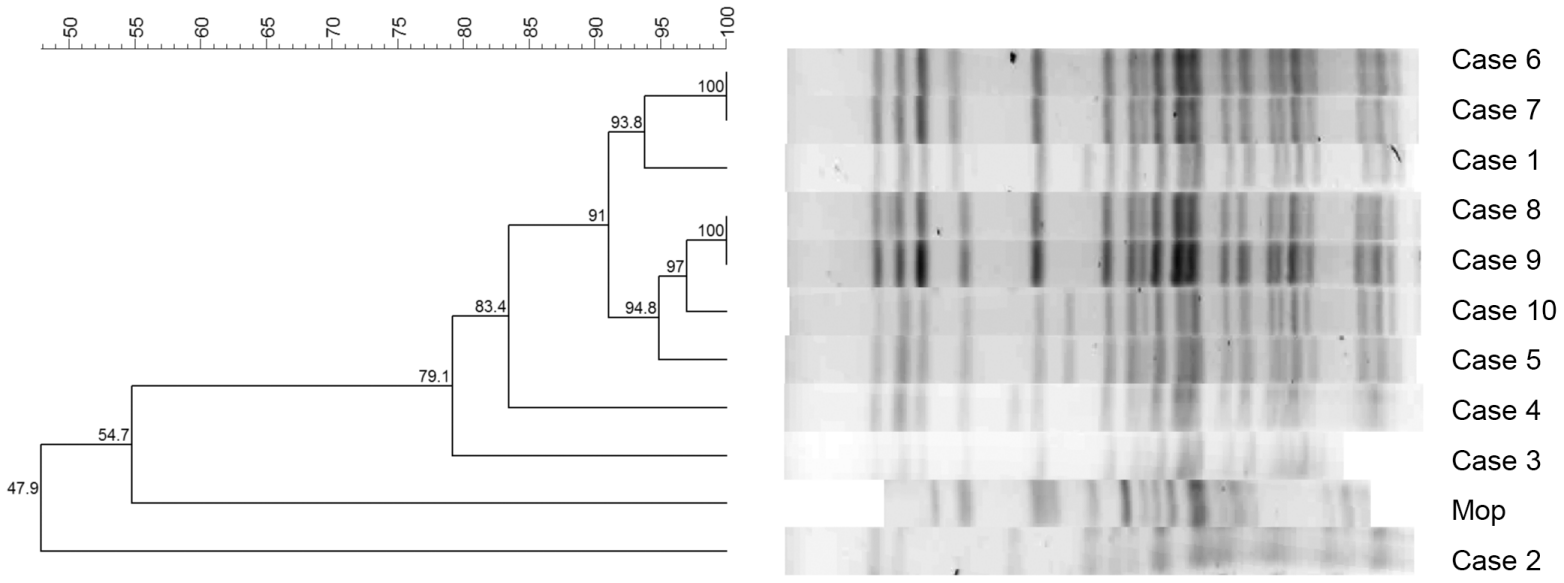
Dendrogram of clinical and environmental *Pseudomonas aeruginosa* isolates from outbreak among patients admitted to the haematology intensive care unit of a tertiary academic hospital in Cape Town, South Africa, January 2010–January 2011.

PFGE analysis shown in Figure 1 revealed that the 9 clinical strains with similar antibiotic resistance profiles were closely related, 8 with greater than 80% similarity, and 1 (Case 3) with 78.9% similarity, while one isolate, Case 2, was unrelated. MLST analysis shown in Table 2 indicated that the isolates from cases 1, 4 and 10 belonged to Sequence Type (ST) 233 while that of Case 2 belonged to ST260 [12] (see Figures S1, S2, S3, S4, S5, S6 and S7 for MLST allele consensus sequences).

**Table 2:** Multi-locus sequence typing (MLST) allelic profiles and sequences for five selected *Pseudomonas aeruginosa* isolates as determined from the MLST database (http://pubmlst.org/paeruginosa/).

	MLST alleles							
Patients	*acs*A	*aro*E	*gua*A	*mut*L	*nuo*D	*pps*A	*trp*E	ST
Case 1	16	5	13	11	4	31	41	233
Case 2	14	5	10	7	4	13	7	260
Case 4	16	5	13	11	4	31	41	233
Case 10	16	5	13	11	4	31	41	233

ST	Sequence Type
*acs*A	Acetyl coenzyme A synthetase
*aro*E	Shikimate dehydrogenase
*gua*A	GMP synthase
*mut*L	DNA mismatch repair protein
*nuo*D	NADH dehydrogenase I chain C, D
*pps*A	Phosphoenolpyruvate synthase
*trp*E	Anthranilate synthetase component I

### Environmental and Staff screening

Twenty six of 51 eligible staff members submitted stool specimens. *P. aeruginosa* was not identified from any of the submitted specimens. Three specimens contained other probable nosocomially acquired organisms: two ESBL-producing *Klebsiella pneumoniae* and one multi-resistant *Acinetobacter baumannii*.

A total of 49 environmental samples and 24 water samples were tested. *P. aeruginosa* was identified in a single specimen taken from a cleaning mop. The isolate was susceptible to ceftazidime and colistin only, and its PFGE profile differed from the patient isolates.

### Case-Control Study

In bivariate analysis MRPA-BSI was associated with hospitalisation longer than 60 days (OR = 60, 95% CI 4.73–2879), AML (OR = 7.8, 95% CI 1.49–51.3) and prior use of amikacin (OR = 0.22, 95% CI 0.04–1.08) and clarithromycin (OR = 12.6, 95% CI 1.16–164.8) when non-GN controls were used. When GN controls were used clarithromycin (OR = 10.7, 95% CI 0.67–584), metronidazole (OR = 16.7, 95% CI 1.2–852) and imipenem (OR = 7.77, 95% CI 1.2–57.8) were significantly associated with MRPA-BSI. (See Table 3)

**Table 3:** Bivariate analysis of risk factors associated with multi-resistant *Pseudomonas aeruginosa* bloodstream infection among patients admitted to the haematology intensive care unit of a tertiary academic hospital in Cape Town, South Africa, January 2010–January 2011.

Risk Factor	Cases	Non-GN Controls		GN Controls			
	(n = 10)	(n = 61)	OR (95% CI)	p-Value	n = 26	OR (95% CI)	p-Value
Female, n (%)	8 (80%)	32 (52.5%)	3.62(0.64–37.11)	0.1036	17 (65.4%)	2.12 (0.311–24.16)	0.3938
**Hospitalisation**							
Public Hospital, n (%)	5 (50%)	32 (52.5%)	0.91 (0.19–4.38)	0.8853	12 (46.1%)	1.17 (0.21–6.45)	0.836
Cumulative length of hospitalisation longer than 2 months (60 days)	5 (50%)	1 (18%)	60 (4.73–2879.6)	<0.0001	8 (30.8%)	2.25 (0.386–12.8)	0.2819
**Diagnosis**							
AML, n (%)	7 (70%)	14 (22.9%)	7.8 (1.49–51.3)	0.0025	13 (50%)	2.33 (0.404–16.7)	0.2794
ALL	1 (10%)	7 (11.5%)	0.86 (0.017–8.17)	0.8912	3 (11.5%)	0.85 (0.015–12.4)	0.8953
Aplastic anaemia	1 (10%)	3 (4.92%)	2.15 (0.037–29.98)	0.5183	1 (3.85%)	2.78 (0.032–226.5)	0.4703
**Therapeutic Procedure**							
Bone Marrow Transplant, n (%)	4 (44.4%)	34 (58.6%)	0.56 (0.102–2.95)	0.4245	9 (37.5%)	1.33 (0.205–8.12)	0.7161
Chemotherapy, n (%)	7 (78%)	32 (55.2)	2.84 (0.48–29.9)	0.2008	18 (75%)	1.17 (0.152–14.46)	0.8683
**Antibiotic use**							
Amikacin, n (%)	4 (40%)	46 (75.4%)	0.22 (0.04–1.08)	0.023	16 (61.5%)	0.42 (0.07–2.33)	0.244
Cotrimoxazole, n (%)	1 (10%)	17 (27.9%)	0.29 (0.006–2.4)	0.2286	0	-	-
Clarithromycin, n (%)	3 (30%)	2 (3.28%)	12.6 (1.16–164.8)	0.0022	1 (3.85%)	10.7 (0.67–584.1)	0.0253
Metronidazole, n (%)	4 (40%)	16 (26.2%)	1.87 (0.34–9.06)	0.3696	1 (3.85%)	16.7 (1.2–852.1)	0.005
Gentamicin, n (%)	2 (20%)	5 (8.19%)	2.8 (0.226–20.8)	0.2458	5 (19.2%)	1.05 (0.084–8.22)	0.9583
Imipenem, n (%)	7 (70%)	38 (62.3%)	1.41 (0.285–9.26)	0.6392	6 (23.1%)	7.77 (1.2–57.8)	0.0087
Ofloxacin, n (%)	7 (70%)	44 (72.1%)	0.901 (0.179–6.03)	0.8896	20 (76.9%)	0.7 (0.109–5.55)	0.6674
Piperacillin/Tazobactam, n (%)	3 (30%)	34 (55.7%)	0.34 (0.053–1.69)	0.131	6 (23.1%)	1.43 (0.18–9.14)	0.6674
Vancomycin, n (%)	6 (60%)	36 (59%)	1.04 (0.22–5.55)	0.9532	11 (42.3%)	2.04 (0.369–12.2)	0.3409

Non-GN Controls: controls who never developed bloodstream infection due to Gram-negative bacteria.

GN Controls: controls who had bloodstream infections due to Gram-negative bacteria other than *P. aeruginosa*.

AML Acute myeloid leukaemia ALL Acute lymphoblastic leukaemia.

Variables independently associated with MRPA-BSI were AML (OR = 19.5, 95% CI 2.03–187, p-value = 0.01) and prior use of amikacin (OR = 0.06, 95% CI 0.006–0.581, p-value = 0.015) when Non-GN controls were used. When GN controls were used, metronidazole (OR = 16.7, 95% 1.56–177, p-value = 0.020) was independently associated with MRPA. (See Table 4)

**Table 4:** Multivariate analysis of risk factors associated with multi-resistant *Pseudomonas aeruginosa* bloodstream infections among patients admitted to the haematology intensive care unit of a tertiary academic hospital, in Cape Town, South Africa, January 2010–January 2011.

Control Group	Risk Factor	Adjusted OR (95% CI)	p-Value
**Non-GN Controls**	AML	19.5 (2.03–187)	0.010
	Amikacin	0.06 (0.006–0.581)	0.015
**GN Controls**	Metronidazole	16.7 (1.56–177)	0.020

AML Acute myeloid leukaemia.

Non-GN Controls patients who never developed bloodstream infection due to Gram-negative bacteria.

GN Controls patients with bloodstream infections due to Gram-negative bacteria other than *P. aeruginosa*.

## Discussion

We report an outbreak of MRPA-BSI infection occurring over a one year period involving 10 immunocompromised patients in a haematology unit of a tertiary hospital in Cape Town. In 9 out of 10 cases the organism was resistant to all antibiotics tested, including carbapenems, and susceptible only to colistin. PFGE analysis showed the 9 isolates to have identical profiles, and this was confirmed with MLST which further indicated that 3 isolates spanning the outbreak period belonged to ST233. Strains belonging to ST233 have been described previously in a Japanese outbreak in 2006–2009 [13] and in a case probably imported to Scandinavia from Ghana [14].

The aim of the outbreak investigation was to identify the source of the outbreak and associated risk factors in order to halt the spread of MRPA in the ward Fortunately, transmission seems to have been halted by the intervention conducted in December 2010 as the only case of MRPA-BSI after the ward was reopened was a patient previously admitted to the ward before December 2010, who was presumed to have become colonised at that stage.

In general, bloodstream infection with *P. aeruginosa* among patients with haematological conditions results chiefly from endogenous sources [15]. The striking similarity among the strains isolated from these cases suggests an exogenous common source. Several potential sources for MRPA may exist in a hospital setting, including colonized patients, staff or the environment.

Transmission of multi-resistant organisms from one patient to another may occur by direct contact or indirectly, with formites or healthcare workers as vectors [16]–[18]. In the current outbreak, direct patient-to-patient transmission was considered unlikely as patients were accommodated in separate isolation rooms and had little or no contact with each other.

Transmission by staff could not be adequately investigated due to the retrospective nature of the investigation. However, screening of staff for long-term gut colonization with MRPA did not reveal any carriers. It should, however, be noted that not all staff participated in the screening initiative. Additionally, the ward has a high turnover of temporary agency nursing staff who could not be accounted for.

Although none of the patients admitted to the haematology ICU between December 2009 and January 2011 showed evidence of gastro-intestinal colonization with MRPA, adherence to the schedule of weekly stool surveillance samples, as specified by the unit protocol, had been inconsistent during this time.

No environmental source of infection was identified. *P. aeruginosa* was isolated from only one of 73 environmental specimens collected. This isolate differed from the outbreak strain in both antibiotic resistance and PFGE profiles. Given the time delay between the occurrence of the first cases in January 2010 and the collection of the environmental samples in October of that year, it is possible that an initial environmental source had existed, but had already been eliminated by the time of the screening. One of the drawbacks of the study was that we were unable to narrow our investigations to the particular isolation rooms occupied by patients with MRPA- BSI, since the unit did not keep records of individual room occupancy.

A case-control study was conducted to identify potential risk factors for *P. aeruginosa* bloodstream infection among patients admitted to the unit. Cases with *P. aeruginosa* BSI were compared to patients with bloodstream infections due to other Gram-negative bacteria and to patients without Gram-negative bloodstream infection. Potential risk factors identified on multivariate analysis included for the comparison to patients with other Gram negative BSIs, the prior use of metronidazole (OR = 16.7, 95% 1.56–177, p-value = 0.020), and for the comparison to patients without other Gram-negative BSIs, an underlying diagnosis of AML (OR = 19.5, 95% CI 2.03–187, p-value = 0.01). The prior use of amikacin (OR = 0.06, 95% CI 0.006–0.581, p-value = 0.015) appeared to be protective in this latter group (See Table 4).

The findings from the case-control study should be interpreted with caution, given the small number of cases, as well as the fact that data for important variables, such as the severity and duration of neutropenia, were not available. The period of observation for controls was generally much longer than the period of observation for cases and thus observational bias may have led to underestimation of certain exposures among the cases. In addition, control group selection in studies of antimicrobial resistant nosocomial pathogens is complicated and use of inappropriate controls may give misleading results [19].

The haematology ward is specifically designed for maximal protection of vulnerable patients; strict infection control protocols are in place and access to the ward is strictly controlled. While it is not unusual for multi-resistant pathogens such as MRPA to be sporadically introduced into such units, the infection control procedures in place should contain the infection. Irrespective of the original source of the MRPA outbreak strain and whether it was subsequently transmitted from human or environmental locations, breaches in infection control may have contributed to the sustained spread of this organism over time.

This outbreak occurred in a shared public–private unit where two separate staff groups share a common environment. There was no evidence to suggest that patients from either the public or private side were more likely to become infected. However, being a shared unit did contribute to the complexity of managing the outbreak in that communication with both sectors was constantly required. The case fatality rate during this outbreak was high in comparison to other studies conducted in similar populations [20]–[22]. This was, in part, due to severe underlying immunocompromise among the patients, but to a larger extent due to extensive resistance of the MRPA strain. Early initiation of empirical therapy with broad-spectrum antibiotics is associated with reduced mortality among patients with neutropenic sepsis [23]–[25]. The MRPA strain encountered in this outbreak was resistant to most antibiotics with antipseudomonal activity and infection progressed so rapidly that even early initiation of colistin therapy in the later cases, did not improve the patient outcomes. Further characterisation of the mechanisms contributing to the multi-resistant phenotype of these strains is ongoing.

Given such a resistant organism and a population in whom infection is so severe and progresses so rapidly, emphasis must be put on preventing acquisition and transmission of infection in the first place. Future efforts on dealing with resistant organisms among this patient population must focus on early identification and decolonization/decontamination of potential sources, possibly using routine screening of patients, staff and the environment as a tool.

Although the haematology unit had a policy of regular screening of patients for gut colonization with resistant pathogens, this had not been implemented consistently in the weeks and months preceding the outbreak. This, coupled with the fact that the outbreak was detected 9 months after the first occurrence, indicates the importance of continuous, consistent surveillance of nosocomial infections among high-risk patients. Surveillance for pathogens in such settings should also incorporate molecular methods to support epidemiological data.

In conclusion, we have described an outbreak of MRPA due to a single strain, but were unable to identify the source of the organism. The outbreak appears to have been contained by the temporary closure and cleaning of the ward in December 2010. Poor adherence to infection control may have facilitated transmission of MRPA. It is vital that in future increased resources and effort is available for the strengthening of infection control practices in the hospital concerned.

Outbreaks of nosocomial infections due to drug resistant organisms are an important infection control problem in both developed and limited-resource countries. They contribute to the overall burden of disease and are often associated with high costs and increased mortality. Despite widespread recognition of drug resistant pathogens as a universal problem, few outbreaks of this nature are investigated and reported in low-resourced countries [26]–[28]. Outbreaks due to drug resistant pathogens may be more likely to occur in middle income countries, where resources allow for the use of broad spectrum antibiotics, but not for adequate infection control and surveillance. As far as we know this is the second published report of a nosocomial outbreak of *P. aeruginosa* from South Africa, the first having been published in 2002 [29]. Our report highlights the problem of antimicrobial-resistant organisms and the infection control challenges that they pose in a limited resource setting. It also highlights the need to create capacity for surveillance and investigation of nosocomial outbreaks.

## Supporting Information

Figure S1 **acsA consensus sequence.**
(TXT)

Figure S2 **aroE consensus sequence.**
(TXT)

Figure S3 **guaA consensus sequence.**
(TXT)

Figure S4 **mutL consensus sequence.**
(TXT)

Figure S5 **nuoD consensus sequence.**
(TXT)

Figure S6 **ppsA consensus sequence.**
(TXT)

Figure S7 **trpE consensus sequence.**
(TXT)
